# The Prevalence of *Arcobacteraceae* in Aquatic Environments: A Systematic Review and Meta-Analysis

**DOI:** 10.3390/pathogens11020244

**Published:** 2022-02-13

**Authors:** Igor Venâncio, Ângelo Luís, Fernanda Domingues, Mónica Oleastro, Luísa Pereira, Susana Ferreira

**Affiliations:** 1 CICS-UBI-Health Sciences Research Centre, University of Beira Interior, 6200-506 Covilhã, Portugal; igorvenancio2012@hotmail.com (I.V.); angelo.luis@fcsaude.ubi.pt (Â.L.); fcd@ubi.pt (F.D.); 2 Grupo de Revisões Sistemáticas (GRUBI), Faculdade de Ciências da Saúde, Universidade da Beira Interior, 6200-506 Covilhã, Portugal; lpereira@ubi.pt; 3 National Reference Laboratory for Gastrointestinal Infections, Department of Infectious Diseases, National Institute of Health Dr. Ricardo Jorge, 1649-016 Lisbon, Portugal; monica.Oleastro@insa.min-saude.pt; 4 CMA-UBI-Centro de Matemática e Aplicações, Universidade da Beira Interior, 6200-001 Covilhã, Portugal; 5 C4-UBI, Cloud Computing Competence Centre, University of Beira Interior, 6200-284 Covilhã, Portugal

**Keywords:** aquatic environment, meta-analysis, *Arcobacteraceae*

## Abstract

Members of the family *Arcobacteraceae* are distributed widely in aquatic environments, and some of its species have been associated with human and animal illness. However, information about the diversity and distribution of *Arcobacteraceae* in different water bodies is still limited. In order to better characterize the health risk posed by members in the family *Arcobacteraceae*, a systematic review and meta-analysis-based method was used to investigate the prevalence of *Arcobacteraceae* species in aquatic environments based on available data published worldwide. The database search was performed using related keywords and considering studies up to February 2021. The pooled prevalence in aquatic environments was 69.2%, ranging from 0.6 to 99.9%. These bacteria have a wide geographical distribution, being found in diverse aquatic environments with the highest prevalence found in raw sewage and wastewater treatment plants (WWTP), followed by seawater, surface water, ground water, processing water from food processing plants and water for human consumption. Assessing the effectiveness of treatments in WWTP in eliminating this contamination, it was found that the wastewater treatment may not be efficient in the removal of *Arcobacteraceae*. Among the analyzed *Arcobacteraceae* species, *Al. butzleri* was the most frequently found species. These results highlight the high prevalence and distribution of *Arcobacteraceae* in different aquatic environments, suggesting a risk to human health. Further, it exposes the importance of identifying and managing the sources of contamination and taking preventive actions to reduce the burden of members of the *Arcobacteraceae* family.

## 1. Introduction

Proposed in 1991, the genus *Arcobacter* was included in the family *Campylobacteracea*, which comprised two more genera, *Campylobacter* and *Sulfurospirillum* [1,2]. Over the years, this genus has been expanded to include more species, currently comprising 34 species, of which 30 are validly published [3,4]. Since the proposal for the creation of the *Arcobacter* genus, it has been subjected to changes, and its taxonomical organization remains controversial. In 2017, after a comparative genomic analysis of the class *Epsilonproteobacteria*, a reclassification of the historically denominated *Arcobacter* genus as a new family denominated *Arcobacteraceae* was proposed to be included in the class *Campylobacteria* [5]. More recently, through phylogenetic and genomic analyses, Pérez-Cataluña et al., (2018) have suggested the reassessment of the taxonomy of genus in order to clarify the relationships among its species. The authors suggested the division of the genus *Arcobacter* in six genera and one candidate [6]. However, despite the reclassification in different genera having been validated [7,8], it has been refuted by considering *Arcobacter* as a taxon phenotypically-, phylogenetically- and genomically-coherent, while accepting the proposal of the family *Arcobacteraceae* as reasonable [9,10]. *Arcobacteraceae* is a family found in diverse habitats, environments and hosts, including animals, humans, foods and food-processing facilities, environmental and water sources such as underground water, surface water, sewage and sea water [11,12,13]. In fact, most of the species from this family have been first isolated from aquatic environments [14]. Few species from this family have been associated with animal and human disease, among which *Aliarcobacter butzleri* and *Aliarcobacter cryaerophilus* have been classified by the International Commission on Microbiological Specification for Food as a serious hazard to human [15]. Despite these two species being the most predominantly associated with human disease, infections with *Aliarcobacter skirrowii*, *Aliarcobacter thereius*, *Malacobacter mytili* and *Aliarcobacter lanthieri* have also been reported [16,17,18,19]. These species can cause intestinal diseases, with symptoms of diarrhea, abdominal pain, nausea, vomiting and fever, but also extraintestinal diseases, such as bacteraemia and peritonitis [20]. Arcobacters are described as water and food-borne bacteria, for which contaminated food or water are considered the probable route of transmission to human and animals [11]. Considering the wide distribution of *Arcobacteraceae* in environmental samples and water sources, the consumption or the direct contact of humans and animals with these bacteria may be seen as a relevant threat to public health. Thus, studies on *Arcobacteraceae* species in water sources can be useful to determine their role as a vehicle for the transmission of infectious agents, ecological characteristics and the potential zoonotic risk of these samples. Although studies on the prevalence of bacteria from this family in different aquatic environments can be found, there is no comprehensive data available on its prevalence to estimate the load. Therefore, the main aim of this work was to perform a systematic review followed by meta-analysis in order to investigate the prevalence of *Arcobacteraceae* in different water bodies based on data available worldwide.

## 2. Results and Discussion

### 2.1. Selection and Characteristics of Studies

**Figure 1:**
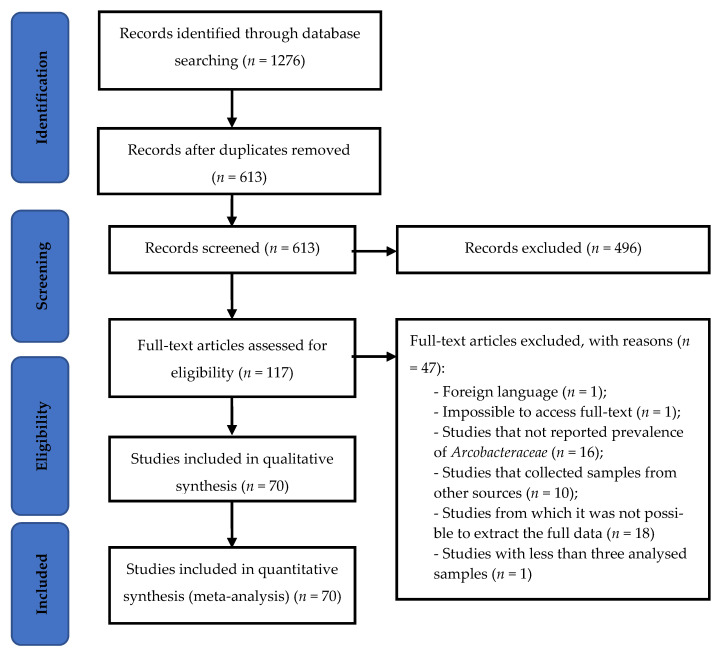
Flow diagram of database search, study selection and articles included in the meta-analysis.

After removing duplicate articles from the searches of the three selected databases, 613 articles were available for title and abstract screening. Of these, 117 were identified as potentially relevant, and 70 were eligible for inclusion after full-text review (Figure 1). The prevalence data was gathered from the articles considering the employment of molecular or cultural methods; when both methodologies were used, the overall value of prevalence was collected and applied for meta-analysis. When it was not possible to recover the full data required for the analysis, the work was not considered, and in the situation of examination of samples from different countries, the study was divided by country. 

### 2.2. Meta-Analysis Results on Overall Prevalence

**Figure 2:**
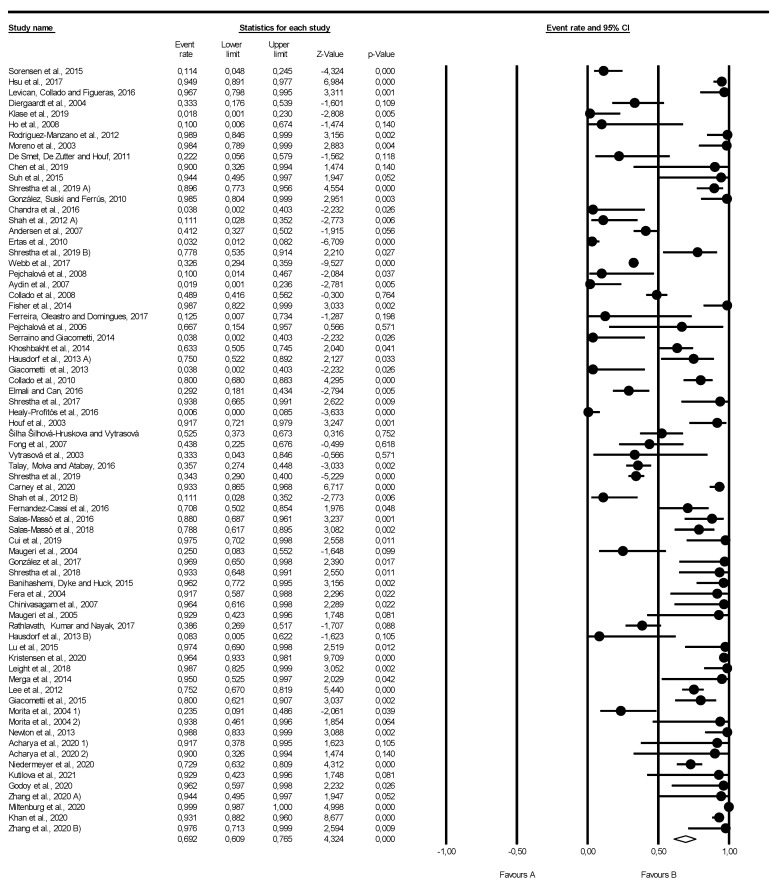
Forest plot of the meta-analysis of prevalence of *Arcobacteraceae* in aquatic environments (in the references, 1 and 2 concern a division of the study by country).

The global prevalence of *Arcobacteraceae* in aquatic environments was investigated considering 70 studies (Figure 2), from which the pooled prevalence was 69.2% (0.692; 95% CI: 0.609–0.765), ranging from 0.6 to 99.9%. The heterogeneity among the studies was found significant, as demonstrated by the values of statistics of the studies included in this meta-analysis (I^2^ = 91.927%; tau^2^ = 1.693; *p*-value < 0.001). The publication bias was assessed by applying a funnel plot generated for the outcome, considering the Trim and Fill adjustment. The adjustment of the funnel plot to the absence of publication bias can be achieved with the inclusion of 8 additional studies (Supplementary Figure S1). The presence of publication bias was further assessed by using Egger’s regression test (Supplementary Table S2). The results of this test showed that there is evidence to reject the null hypothesis (*p*-value < 0.001), indicating that there is asymmetry in the funnel plot. Consequently, apparent publication bias exists in the studies included in this meta-analysis, which can be justified by the relevance of the publication of articles with positive results regarding the presence of *Arcobacteraceae* in aquatic environment samples.

### 2.3. Subgroup Analysis by Geographical Distribution

A subgroup analysis based on the country and continent of origin was taken (Table 1). *Arcobacteraceae* in aquatic environments have been reported in 26 countries among the 70 included papers. However, a small number of surveys in water were conducted in each of the 26 different countries considered, usually with a low number of samples (from three to 780, with a median number of 24 samples). Country-level estimates showed that the highest pooled prevalence of *Arcobacteraceae* can be found in Denmark, followed by Brazil, Australia and Korea, while the lowest prevalence was observed for the Netherlands and Cameroon. 

Regarding the prevalence of *Arcobacteraceae* among continents, South America showed the highest pooled prevalence with 96.2% (0.962; 95% CI: 0.350–0.999), followed by Oceania, North America, Europe, Asia and finally Africa, with the lowest pooled prevalence value of 19.2% (0.192; 95% CI: 0.047–0.536) (Table 1).

When analyzing prevalence data in subgroups categorized by the income level of the countries, the highest prevalence was presented by countries with a low-income level, at 90.0% (0.900; 95% CI: 0.145–0.998), followed by the countries of high-income level, at 79.0% (0.79; 95% CI: 0.702–0.858), of upper middle-income level, at 47.2% (0.472; 95% CI: 0.281–0.673) and lower middle, at 39.8% (0.398; 95% CI: 0.180–0.666). A high heterogeneity (I^2^ > 75%) was observed in the subgroup analysis by countries, except for Czech Republic and India, which showed a moderate heterogeneity. Additionally, the I^2^ statistics demonstrated a high heterogeneity (I^2^ > 75%) for all the continents. This parameter was not calculated when less than three studies were considered.

Industrial pollution load, poor water and sewage treatment facilities, inadequate water pollution control laws and rapid urbanization rates have contributed to the increasing degradation of the aquatic environment in many developed and developing countries [21], which in turn may potentiate the emergence of genus *Arcobacteraceae*. The observed scenario regarding the prevalence according to the geographical location and level of economic development must be analyzed carefully, given the high heterogeneity between studies. The estimated prevalence by geographical location is clearly affected by the type of samples analyzed in each study, as all types of aquatic samples were included in this analysis, namely from wastewater treatment plants. Nonetheless, this analysis shows the global distribution of members of the family *Arcobacteraceae* in aquatic environments worldwide. Furthermore, several other parameters may influence the observed trend, such as the methods of detection used among studies and even the distribution of studies analyzed by countries. Indeed, the highest prevalence was observed for South America and for the low-income country included that considered only one study, with a low number of samples analyzed (twelve and four samples, respectively). Nonetheless, in general, more studies are globally needed to understand the prevalence of *Arcobacteraceae* worldwide.

### 2.4. Subgroup Analysis by Parameters of Samples Analysis

As the volume of the sample analyzed is a parameter that may clearly influence the prevalence of *Arcobacteraceae*, we further performed a subgroup analysis considering the sample size. For that, the studies were divided into four groups, regarding the amount of sample analyzed (Volume of sample 0–200 mL; 201–500 mL; 1 L and >1 L). When it was not possible to clearly define the volume of sample used in the analysis, the studies were excluded. Through the analysis of the results, it was observed that when the volume of the samples was up to 200 mL, there was a lower prevalence of detection of *Arcobacteraceae* (58.7%), but if the volume exceeded 201 mL, the estimated prevalence increased to at least 82.0% (Table 2).

The amount of used sample is one of the factors that may influence the isolation or detection of bacteria. When a bacterium is present in a low number in environmental water samples, the straightforward way is to analyze larger sample volumes to increase the recovery; however, in turbid environmental water, for example, the high levels of background bacteria can interfere and prevent the isolation or detection of bacteria, such as described for thermotolerant campylobacters [22]. In the case of bacteria from the *Arcobacteraceae* family, the influence of the volume of sample has not been clarified, with only a limited number of the studies examining its presence using a quantitative approach. 

In addition to the volume of sample analyzed, the laboratory detection technique used will likely influence the reported prevalence. Herein, data was divided and analyzed considering five subgroups (Table 3). Considering the results, studies using molecular methodologies presented a higher estimated prevalence when compared with culture techniques. Furthermore, similar prevalence values were found for direct and after enrichment isolation, or for direct or after enrichment molecular detection, when excluding the metagenomic studies. The use of molecular methods allows a faster and more sensitive detection of bacteria, being able to detect both viable and non-viable cells, as well as viable but not cultivable cells. Nonetheless, this methodology has some drawbacks as well, associated with the fact that some molecular methodologies do not allow to distinguish dead from live cells or to recover bacterial isolates that can be used for further studies [14,23,24,25]. Several culture methods are used and the recovery of bacteria from this family can be associated with various factors related with the sample, but also with the disparity in the sensitivity and specificity of isolation methods [25], pointing out the need for a standard protocol for the isolation of *Arcobacteraceae* species from diverse samples [11,23,25]. Also, the use of a selective supplements may lead to lower recovery rates in environmental water samples, due to stressed or injured cells, which may be affected by using these compounds leading to a reduced recovery rate [25,26]. Despite this, when data from culture methodologies with or without an enrichment step are examined, prevalence estimates are close to 43.3% (0.433; 95% CI: 0.348–0.521) and 48.7% (0.487; 95% CI: 0.274–0.705), respectively. Considering the results related to direct molecular detection, a subgroup analysis was performed, dividing data into detection by metagenomic sequencing methodologies and detection methods by PCR techniques or other methods of nucleic acids amplification. When comparing these methodologies, the highest percentage of the detection of *Arcobacteraceae* species was achieved through direct sequencing of the samples (96.0%) instead of using conventional PCR identification techniques (68.8%). This may be associated with the fact that most of PCR methods are directed for some species-specific detection, which has intrinsic limitations beyond the ones associated with the methodology used.

Considering the diversity of protocols used for the isolation, detection and identification of *Arcobacteraceae* members, these data must be interpreted with caution.

### 2.5. Subgroup Analysis by Aquatic Source

A subgroup analysis based on the type of sample examined was performed, taking into consideration the wastewater treatment plants (WWTP) at three distinct stages: influent, treatment at any point and effluent. The results showed that the pooled prevalence of samples collected from raw sewage and WWTP were the ones with the highest prevalence values, followed by samples of seawater, surface water, ground water, processing water from food processing plants and, lastly, water classified as for human consumption with a prevalence of 3.2% (Table 4). The high values of pooled prevalence found in seawater and surface water may be a concern due to its potential recreational use, but also due to its possible influence on the food chain. Further, surface and groundwater are usually used as a water source in developing countries for multiple purposes, increasing the potential health risk. In turn, the lower values of the estimated prevalence of *Arcobacteraceae* species in processing water and drinking water may be associated with the potential inactivation effect of these bacteria by the chlorination process of the water [27], which may be ineffective [28,29]. 

The presence of *Arcobacteraceae* in environmental waters indicates that it can survive and persist in those waters, which points to their potential to be waterborne pathogens. Furthermore, water can act as a contamination vehicle for these species, namely in the food chain [3].

Some studies suggest that fecal contamination may be responsible for introducing these bacteria into the water, being the presence of arcobacters correlated with a high level of fecal pollution [30]. In fact, among the outbreaks associated with arcobacters, some have suggested that the consumed water could have been contaminated by sewage [31,32]. However, the presence of high recovery rates of *Arcobacteraceae* in sea and surface waters may be due not only to the proximity of possible sources of fecal pollution, but also because several species have already been described as native to marine environments. In fact, many waterborne species of this family are found with high frequency in seawater or seafood from coastal waters [3,33]. 

The highest prevalence in this meta-analysis was found in the wastewater entering wastewater treatment plants (WWTP), which is in line with the reported prevalence in raw sewage. Thus, to assess the effectiveness of treatments in WWTP in eliminating this contamination, we followed with a subgroup analysis. Despite that a small decrease in the pooled prevalence of *Arcobacteraceae* through the WWTP was observed, a high prevalence continues to be observed, which could be seen as a potential health concern. Some authors suggest that these species are well suited to survive in adverse conditions, such as those in wastewater treatment plants, where their discharge into the environment is classified as a global problem [34]. Kristensen et al., 2020 described that the high relative abundance of arcobacters in the effluent may be associated with the removal of influent microorganisms in biological WWTPs. In the case of arcobacters, a large fraction of cells dispersed in the water phase prevails due to the reduced ability of these bacteria to flocculate and attach to the activated sludge flocs, preventing their effective removal [34]. This points to the need to reevaluate the treatment processes adopted or to even develop more effective methodologies to eliminate or potentially minimize the discharge of this emerging pathogen.

### 2.6. Subgroup Analysis by Arcobacteraceae Species

Considering that species from *Arcobacteraceae* family can be seen as waterborne pathogens, but also as naturally found in these environments, we proceeded with a subgroup analysis considering the different species. In this subgroup analysis, when a study presented prevalence data for each species determined by culture and molecular techniques, the global value or the highest value was collected for analysis. When evaluating the prevalence of the different species identified in the several categories of water samples, considering the ones that were identified in at least three studies, *Al. butzleri* was the species with the highest overall prevalence (58.3%), followed by *Al. cryaerophilus* (42.5%), *Malaciobacter mytili* (16.2%), *Al. thereius* (15.4%), *Pseudarcobacter cloacae* (14.8%), *Pseudarcobacter defluvii* (14.7%), *Al. skirrowii* (12.7%) and *Arcobacter nitrofigilis* (8.8%) (Table 5). *Al. butzleri* presented the highest pooled prevalence in most different water categories revealing the highest prevalence in seven of nine different water types, followed by *Al. cryaerophilus*, two of the species most associated with human diseases.

Çelik and Ünver (2005) suggested that *Al. butzleri* may present a stronger viability than other species in water, while presenting a competitive inhibitory effect in the population dynamic with other species [35]. Nonetheless, when analyzing these results, it should be considered that isolation and identification methods are needed for the analysis of the species considered, since the currently used methodologies may lead to an underestimation of the presence of some *Arcobacteraceae* species throughout the aquatic environment. 

This systematic review and meta-analysis on the prevalence of species from the *Arcobacteraceae* family provides a comprehensive analysis on its occurrence and wide distribution worldwide. The use of meta-analytic techniques to assess the prevalence of pathogens in the environment, while allowing to overcome some flaws of the traditional review, also has the advantage of considering the relative weight with which each individual study contributes to the final result. The lack of defined criteria for carrying out the systematic review and meta-analysis outside clinical settings represents, however, one of its weaknesses. In addition, this study also includes some limitations: (a) there was a lack of studies in some regions across the world, (b) the confounding effect of using samples from different aquatic environments in the global estimate of prevalence, (c) the low number of samples analyzed in some studies or (d) the diversity of the detection and identification methods with different sensitivities and specificities.

Despite this, the results confirm that the species from this family have a wide geographical distribution, being present on diverse aquatic environments. The presence of the pathogenic species in these environments represents a public health risk, particularly when accessible to animals and humans. Thus, this study demonstrates the need for the monitoring and surveillance of water quality and safety, considering the presence of *Arcobacteraceae* species, as well as to assess to microbial risk. Further, some concern can be associated with *Arcobacteraceae* in wastewater plants effluents, highlighting the need for rapid action and review of bacterial elimination processes of this family, as effluents may eventually impact the receiving water body quality and in turn contaminate animals and food products that are easily accessible to humans. 

## 3. Materials and Methods

### 3.1. Search Strategy and Study Selection

A comprehensive systematic literature search from databases ISI Web of Science, PubMed and Scopus were accessed for studies in February 2021 using the following search strategy: Arcobacter AND (Water OR groundwater OR seawater OR influent OR Effluent OR ambient OR sewage OR wastewater). This systematic review was performed following the PRISMA protocol. The recovered records were exported to Rayyan QCRI (https://rayyan.qcri.org/welcome (accessed on 31 January 2022)) for the initial screening. Thereafter, all the studies from the search were independently analyzed by the title, abstract and selected full-text by two reviewers, and a third reviewer arbitrated any divergence. Full-text articles published or in press were collected, while reviews, conference abstracts and chapter books were excluded. Only studies in English, Portuguese and Spanish were accessed for inclusion. Articles were considered for full-text review if (1) the full-text article could be retrieved, (2) it reported primary data or (3) the article reported isolation by culture or detection by molecular techniques of *Arcobacteraceae* or their species in water samples. 

### 3.2. Data Extraction and Statistical Analyses

After a careful analysis, the following data were extracted and summarized from each included article: first author’s last name, year of publication, country, continent, income level, total analyzed samples, source of the samples, detection technique, volume of water used for analysis, species identified/detected and prevalence or number of positive samples. Meta-analysis of the prevalence of *Arcobacteraceae* was performed using Comprehensive Meta-Analysis Software v.2.0 (https://www.meta-analysis.com/ (accessed on 31 January 2022)). Forest plots were generated to show the study-specific effect sizes, with the pooled prevalence (PP) considered with a 95% confidence interval (CI), using the random-effects model. Heterogeneity among studies was measured by applying the I^2^ statistics. Values close to 0% indicate no heterogeneity, whilst values close to 25%, 50% and 75% correspond to a low, moderate and high heterogeneity, respectively. *p*-values correspond to the heterogeneities between studies from a Chi-squared test of the null hypothesis that there is no heterogeneity. The potential impact of publication bias on the present meta-analysis was assessed by three different analyses: funnel plot [36,37]; Egger’s regression test [38,39] and Duval and Tweedie’s Trim and Fill approach [40,41]. This allowed us to obtain the best estimate of the unbiased pooled effect size, creating a funnel plot including both the observed studies (shown as blue circles) and the necessary imputed studies (shown as red circles) to obtain the absence of bias. A sensitivity analysis was performed by removing each study at a time to evaluate the stability of the results. Subgroup analysis was performed on the outcome under the study per countries, continents (Turkey was included in Asia), income level, volume of analyzed water, laboratory detection technique, *Arcobacteraceae* species and water types.

## Figures and Tables

**Table 1:** Meta-analysis of the prevalence of *Arcobacteraceae* according to countries, continents and income level.

Countries/Continent/Income Level	*n*	Pooled Prevalence	95% CI		Q-Value	I^2^	tau^2^	*p*-Value
			Lower Limit	Upper Limit				
Countries								
Australia	2	0.946	0.607	0.995	0.2	0	0	0.655
Belgium	2	0.647	0.15	0.95	11.215	91.084	6.069	0.001
Brazil	1	0.962	0.298	0.999	0	0	0	1
Cameroon	1	0.006	0	0.248	0	0	0	1
Canada	4	0.919	0.686	0.983	157.866	98.1	6.533	0
China	6	0.88	0.582	0.975	22.112	77.388	7.142	<0.001
Czech Republic	5	0.495	0.164	0.83	8.137	50.839	0.886	0.087
Denmark	1	0.964	0.571	0.998	0	0	0	1
Ethiopia	1	0.9	0.125	0.998	0	0	0	1
Germany	2	0.458	0.066	0.91	4.993	79.972	4.889	0.025
India	2	0.194	0.021	0.728	3.523	71.611	2.717	0.061
Iran	1	0.633	0.08	0.972	0	0	0	1
Italy	6	0.496	0.184	0.811	28.095	82.203	3.702	<0.001
Japan	1	0.235	0.091	0.486	0	0	0	1
Korea	1	0.944	0.221	0.999	0	0	0	1
Malaysia	2	0.111	0.012	0.56	0	0	0	1
Nepal	4	0.78	0.408	0.948	29.843	89.947	2.843	<0.001
Netherlands	1	0.1	0.002	0.875	0	0	0	1
Portugal	1	0.125	0.002	0.903	0	0	0	1
South Africa	1	0.333	0.023	0.914	0	0	0	1
Spain	10	0.894	0.742	0.961	63.897	85.915	1.383	<0.001
Thailand	1	0.938	0.461	0.996	0	0	0	1
Turkey	4	0.124	0.028	0.413	31.328	90.424	1.365	<0.001
UK	2	0.935	0.443	0.996	0.07	0	0	0.792
USA	9	0.857	0.669	0.947	98.424	91.872	1.361	0
Zambia	1	0.114	0.006	0.736	0	0	0	1
Continent								
Africa	4	0.192	0.047	0.536	17.351	82.71	2.322	<0.001
Asia	22	0.499	0.336	0.663	149.357	85.939	1.143	0
Europe	30	0.727	0.596	0.828	193.062	84.979	1.846	0
North America	13	0.871	0.749	0.939	348.869	96.56	2.16	0
Oceania	2	0.945	0.654	0.994	0.2	0	0	0.655
South America	1	0.962	0.35	0.999	0	0	0	1
Income level								
Low	1	0.9	0.145	0.998	0	0	0	1
Lower middle	8	0.398	0.18	0.666	54.123	87.067	1.211	<0.001
Upper middle	16	0.472	0.281	0.673	116.089	87.079	1.95	0
High	47	0.79	0.702	0.858	644.845	92.867	1.956	0

**Table 2:** Meta-analysis of the prevalence of *Arcobacteraceae* according to the volume of sample analyzed.

Volume of Sample (mL)	*n*	Pooled Prevalence	95% CI		Q-Value	I^2^	tau^2^	*p*-Value
			Lower Limit	Upper Limit				
0–200	44	0.587	0.479	0.687	445.848	90.356	1.312	<0.001
201–500	4	0.820	0.480	0.958	32.095	90.653	10.209	<0.001
1000	3	0.903	0.684	0.976	8.358	76.069	0.517	<0.001
>1000	7	0.858	0.666	0.948	72.324	91.704	4.86	<0.001

**Table 3:** Meta-analysis of the prevalence of *Arcobacteraceae* according to the used detection method.

Methods	*n*	Pooled Prevalence	95% CI		Q-Value	I^2^	tau^2^	*p*-Value
			Lower Limit	Upper Limit				
Culture—after enrichment	37	0.433	0.348	0.521	278.038	87.052	0.779	<0.001
Culture—without enrichment	4	0.487	0.274	0.705	9.535	68.536	0.543	0.023
Molecular after enrichment	10	0.631	0.437	0.79	39.809	77.392	0.924	<0.001
Molecular direct	30	0.876	0.769	0.937	422.349	93.134	4.281	<0.001
*—metagenomic sequencing*	16	0.96	0.891	0.986	7.954	0	0	0.926
*—PCR and other amplification methods*	14	0.688	0.438	0.862	277.222	95.311	4.058	<0.001

**Table 4:** Meta-analysis of the prevalence of *Arcobacteraceae* according to sample sources.

Sources Samples	*n*	Pooled Prevalence	95% CI		Q-Value	I^2^	tau^2^	*p*-Value
			Lower Limit	Upper Limit				
Seawater	11	0.780	0.600	0.893	93.283	89.280	1.453	<0.001
Surface water	28	0.645	0.485	0.778	368.525	92.673	2.406	<0.001
Ground water	7	0.396	0.198	0.636	31.59	81.007	1.176	<0.001
Raw sewage	20	0.906	0.786	0.962	120.608	84.246	3.325	<0.001
Processing Water	9	0.343	0.141	0.624	33.624	76.207	1.942	<0.001
Drinking water	7	0.032	0.014	0.069	2.791	0	0	0.835
WWTP								
Influent WWTP Treatment WWTP	11	0.964	0.93	0.982	3.556	0	0	0.965
	7	0.931	0.752	0.984	24.559	75.569	2.703	<0.001
Effluent WWTP	9	0.876	0.774	0.936	12.725	37.13	0.366	0.122

**Table 5:** Meta-analysis of the prevalence of *Arcobacteraceae* according to the sources of samples and species.

Species	Drinking Water, Animals		Drinking Water, Humans		Surface Water		Seawater		Processing Water		Raw Sewage		Influent WWTP		Treatment WWTP		Efluent WWTP		Overall	
	*n*	Pooled Prevalence (95% CI)	*n*	Pooled Prevalence (95% CI)	*n*	Pooled Prevalence (95% CI)	*n*	Pooled Prevalence (95% CI)	*n*	Pooled Prevalence (95% CI)	*n*	Pooled Prevalence (95% CI)	*n*	Pooled Prevalence (95% CI)	*n*	Pooled Prevalence (95% CI)	*n*	Pooled Prevalence (95% CI)	*n*	Pooled Prevalence (95% CI)
*Aliarcobacter butzleri*	3	0.090 (0.019–0.342)	2	0.029 (0.004–0.195)	18	0.503 (0.347–0.659)	5	0.704 (0.389–0.898)	3	0.090 (0.019–0.342)	13	0.696 (0.502–0.838)	4	0.954 (0.776–0.992)	3	0.832 (0.485–0.963)	3	0.830 (0.49–0.961)	56	0.583 (0.483–0.675)
*Aliarcobacter skirrowii*	0	-̵̵̵̵̶-	0	-̵̵̵̵̶-	1	0.962 (0.597–0.998)	1	0.042 (0.003–0.425)	0	-̵̵̵̵̶-	1	0.033 (0.062–0.366)	1	0.125 (0.031–0.386)	3	0.071 (0.014–0.288)	1	0.071 (0.004–0.577)	8	0.127 (0.057–0.258)
*Aliarcobacter cryaerophilus*	2	0.079 (0.007–0.50)	0	-̵̵̵̵̶-	4	0.465 (0.120–0.847)	4	0.207 (0.049–0.570)	2	0.089 (0.01–0.479)	9	0.500 (0.251–0.749)	3	0.524 (0.135–0.885)	4	0.770 (0.369–0.950)	2	0.796 (0.237–0.98)	30	0.425 (0.285–0.579)
*Aliarcobacter thereius*	0	-̵̵̵̵̶-	0	-̵̵̵̵̶-	0	-̵̵̵̵̶-	0	-̵̵̵̵̶-	0	-̵̵̵̵̶-	1	0.167 (0.023–0.631)	1	0.167 (0.023–0.631)	3	0.167 (0.055–0.409)	1	0.071 (0.004–0.577)	6	0.154 (0.068–0.311)
*Arcobacter nitrofigilis*	0	-̵̵̵̵̶-	0	-̵̵̵̵̶-	0	-̵̵̵̵̶-	1	0.042 (0.003–0.425)	0	-̵̵̵̵̶-	2	0.108 (0.027–0.346)	1	0.167 (0.023–0.631)	3	0.071 (0.014–0.288)	3	0.071 (0.014–0.288)	10	0.088 (0.041–0.178)
*Malaciobacter mytili*	0	-̵̵̵̵̶-	0	-̵̵̵̵̶-	0	-̵̵̵̵̶-	2	0.207 (0.062–0.506)	0	-̵̵̵̵̶-	1	0.033 (0.001–0.475)	0	-̵̵̵̵̶-	0	-̵̵̵̵̶-	0	-̵̵̵̵̶-	3	0.162 (0.052–0.405)
*Pseudarcobacter* *cloacae*	0	-̵̵̵̵̶-	0	-̵̵̵̵̶-	0	-̵̵̵̵̶-	1	0.091 (0.013–0.439)		-̵̵̵̵̶-	2	0.197 (0.054–0.512)	1	0.333 (0.084–0.732)	3	0.071 (0.014–0.288)	1	0.071 (0.004–0.577)	8	0.148 (0.072–0.281)
*Pseudarcobacter defluvii*	0	-̵̵̵̵̶-	0	-̵̵̵̵̶-	0	-̵̵̵̵̶-	1	0.042 (0.003–0.425)	0	-̵̵̵̵̶-	2	0.150 (0.049–0.377)	1	0.167 (0.023–0.631)	3	0.167 (0.055–0.409)	2	0.160 (0.031–0.530)	9	0.147 (0.078–0.261)

-̵̵̵̵̶-: Corresponds to no value.

## Data Availability

Data are contained within the text and the Supplementary Materials.

## Supplementary Materials

The following are available online at https://www.mdpi.com/article/10.3390/pathogens11020244/s1, Figure S1: Funnel plot of standard error by logit event rate (publication bias tests) for Arcobacteraceae prevalence in aquatic environments, Table S1: Main characteristics of the 70 included studies in this meta-analysis. and Table S2: Assessment of publication bias for the prevalence of Arcobacteraceae in aquatic environments using Egger’s regression test [24,26,28,30,31,33,34,42,43,44,45,46,47,48,49,50,51,52,53,54,55,56,57,58,59,60,61,62,63,64,65,66,67,68,69,70,71,72,73,74,75,76,77,78,79,80,81,82,83,84,85,86,87,88,89,90,91,92,93,94,95,96,97,98,99,100,101,102,103].
