# Supplementary material for: The Prevalence of Arcobacteraceae in Aquatic Environments: A Systematic Review and Meta-Analysis
Source: Pathogens. 2022 Feb 13;11(2):244. doi: 10.3390/pathogens11020244 (PMC8880612; doi:10.3390/pathogens11020244)
Supplement: Supplementary file 1 [file pathogens-11-00244-s001.zip › pathogens-1565023-SM.pdf]

Supplementary Materials

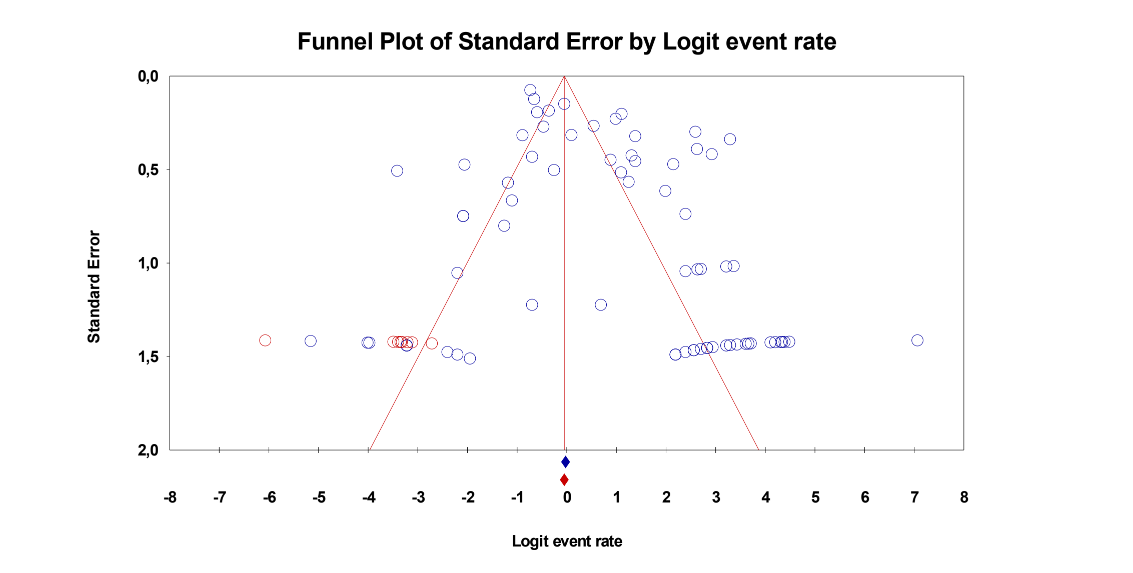

Figure S1. Funnel plot of standard error by logit event rate (publication bias tests) for *Arcobacteraceae* prevalence in aquatic environments.

Table S1. Main characteristics of the included studies in this meta-analysis.

Authors	Year	Country	Continent	Income level	Total analyzed samples	Source of the samples	Detection technique	Volume of water
Sorensen et al. [42]	2015	Zambia	Africa	Lower middle	44	Ground water	Molecular – direct	1-2 L
Hsu et al. [43]	2017	USA	North America	High	118	Wetland water	Molecular – direct	1 L
Levican, Collado and Figueras [44]	2016	Spain	Europe	High	30	Influent and effluent from WWTP	Culture with and without enrichment; Molecular with enrichment; Molecular – direct	200 mL or 400 µL when direct PCR
Diergaardt et al. [45]	2004	South Africa	Africa	Upper middle	24	Surface and ground waters, raw sewage, tap water	Culture with enrichment	-
Klase et al. [46]	2019	China	Asia	Upper middle	27	Water from fishponds	Molecular – direct	200 mL
Ho et al. [47]	2008	Netherlands	Europe	High	4	Tap water from slaughterhouse	Culture with enrichment	50 mL
Rodriguez-Manzano et al. [48]	2012	Spain	Europe	High	44	Wastewater from a WWTP (influent - raw sewage)	Molecular – direct	-
Moreno et al. [49]	2003	Spain	Europe	High	30	River water, influent and effluent from WWTP	Culture and molecular with enrichment	300 mL without enrichment, 100 mL with enrichment
De Smet, De Zutter and Houf [50]	2011	Belgium	Europe	High	9	Non-chlorinated drinking water (from sheep and/or goat farms)	Culture with enrichment	100 mL
Chen et al. [51]	2019	China	Asia	Upper middle	4	Wastewater, effluent from WWTP	Molecular – direct	-
Suh et al. [52]	2015	South Korea	Asia	High	8	Surface seawater	Molecular – direct	30 L
Shrestha et al. [101]	2019	USA	North America	High	48	River water, influent and effluent from WWTP	Molecular – direct	1.1 L

González, Suski and Ferrús [54]	2010	Spain	Europe	High	33	Influent from WWTP	Culture with and without enrichment; Molecular with enrichment; Molecular – direct	100 mL
Chandra et al. [55]	2016	India	Asia	Lower middle	12	Drinking water (household taps)	Molecular – direct	2 L
Shah et al. [102]	2012	Malaysia	Asia	Upper middle	18	Drinking water from animal farm	Culture with enrichment	20 mL
Andersen et al. [57]	2007	USA	North America	High	119	Drinking water (drinkers in farms)	Culture with enrichment	1 mL
Ertas et al. [28]	2010	Turkey	Asia	Upper middle	125	Spring water, drinking water (chlorinated)	Culture with enrichment	20 mL
Shrestha et al. [53]	2019	Nepal	Asia	Lower middle	18	Groundwater	Molecular – direct	10 mL
Webb et al. [58]	2017	Canada	North America	High	780	Surface water, treated wastewater from WWTP	Culture with enrichment	100–250 mL
Pejchalová et al. [59]	2008	Czech Republic	Europe	High	10	-	Culture with enrichment	200 mL
Aydin et al. [60]	2007	Turkey	Asia	Upper middle	26	Drinking water (house tap water)	Culture with enrichment	20 mL
Collado et al. [30]	2008	Spain	Europe	High	178	Seawater, river and lake waters, sewage	Culture with enrichment	200 mL
Fisher et al. [61]	2014	USA	North America	High	37	Influent from WWTP	Molecular – direct	25 mL
Ferreira, Oleastro and Domingues [62]	2017	Portugal	Europe	High	3	Drinking water (tap water from dairy plant)	Culture and molecular with enrichment	10 mL
Pejchalová et al. [103]	2006	Czech Republic	Europe	High	3	Surface water	Culture with enrichment	10 mL
Serraino and Giacometti [63]	2014	Italy	Europe	High	12	Processing water from dairy plants	Culture with enrichment	25 mL
Khoshbakht et al. [64]	2014	Iran	Asia	Upper middle	60	Chiller tank and processing waters from broilers' slaughterhouse,	Molecular with enrichment	1 mL
Hausdorf et al. [65]	2013	Germany	Europe	High	20	Tap water, water from wash basins of a spinach processing plant and water	Molecular with and without enrichment	1 L

						from blancher of a spinach processing plant		
Giacometti et al. [66]	2013	Italy	Europe	High	12	Tap water from a dairy plant	Culture with enrichment	25 mL
Collado et al. [67]	2010	Spain	Europe	High	60	River water, influent and effluent from WWTP and sewage, semi-treated drinking water (at a treatment plant)	Culture with enrichment	200 mL
Elmali and Can [68]	2016	Turkey	Asia	Upper middle	48	Slaughterhouse wastewater	Culture with enrichment	20 mL
Shrestha et al. [69]	2017	Nepal	Asia	Lower middle	16	Spring and river waters, shallow dug wells and deep tube wells	Molecular – direct	100 mL
Healy-Profitós et al. [70]	2016	Cameroon	Africa	Lower middle	86	Drinking water (water storage containers and drinking water sources of households)	Molecular – direct	300 to 750 mL
Houf et al. [71]	2003	Belgium	Europe	High	24	Processing water from a poultry slaughterhouse	Culture with enrichment	50 mL
Šilha, Šilhová-Hruskova and Vytřasová [72]	2015	Czech Republic	Europe	High	40	River and pond waters, well water, wastewater	Culture with enrichment	100 mL
Fong et al. [31]	2007	USA	North America	High	16	Ground water	Culture without enrichment	-
Vytřasová et al. [73]	2003	Czech Republic	Europe	High	3	River and pond waters	Culture with enrichment	-
Morita et al. [74]	2004	Japan and Thailand	Asia	High/Upper middle	24	River and canal waters	Culture with enrichment	50 mL
Talay, Molva and Atabay [26]	2016	Turkey	Asia	Upper middle	115	River and spring waters, sewage, drinking water	Culture with enrichment	200 mL
Shrestha et al. [75]	2019	Nepal	Asia	Lower middle	286	Surface water	Molecular – direct	10–100 mL
Carney et al. [76]	2020	Australia	Oceania	High	104	Seawater	Molecular – direct	2.5 L

Shah et al. [56]	2012	Malaysia	Asia	Upper middle	18	Treated (chlorinated) water from outlets of water supply tank at farms	Culture with enrichment	10 mL
Fernandez-Cassi et al. [77]	2016	Spain	Europe	High	24	Effluent from the secondary outlet of the WWTP as it entered the lagooning system	Culture with enrichment	0.5 mL
Salas-Massó et al. [78]	2016	Spain	Europe	High	25	Marine water, channel of untreated sewage that drains in marine water	Culture with enrichment	200 mL
Salas-Massó et al. [33]	2018	Spain	Europe	High	33	River water, sewage	Culture with enrichment	200 mL
Cui et al. [79]	2019	China	Asia	Upper middle	19	River water, sewage	Molecular – direct	100 mL/0.9–3.4 L/1–2.5 L
Maugeri et al. [80]	2004	Italy	Europe	High	12	Seawater	Culture with enrichment	-
González et al. [81]	2007	Spain	Europe	High	15	Wastewater	Culture and molecular with enrichment	25 mL
Shrestha et al. [82]	2018	Nepal	Asia	Lower middle	15	River water, deep tube well and shallow dug well waters	Molecular – direct	100 mL
Banihashemi, Dyke and Huck [83]	2015	Canada	North America	High	26	River water	Molecular – direct	2 L
Fera et al. [24]	2004	Italy	Europe	High	12	Seawater	Culture with enrichment; Molecular – direct	3 L
Chinivasagam et al. [84]	2007	Australia	Oceania	High	13	Effluent piggeries	Culture with enrichment	10 mL
Maugeri et al. [80]	2005	Italy	Europe	High	6	Seawater	Culture and molecular with enrichment	10 mL
Rathlavath, Kumar & Nayak [85]	2017	India	Asia	Lower middle	57	Coastal water	Culture and molecular with enrichment	1 mL
Hausdorf et al. [86]	2013	Germany	Europe	High	5	Tap water, water from wash basins of a spinach processing plant and water from blancher of a spinach processing plant	Culture without enrichment	50 mL

Lu et al. [87]	2015	China	Asia	Upper middle	18	-	Molecular – direct	250–300 mL
Kristensen et al. [34]	2020	Denmark	Europe	High	252	Influent and effluent WWTP	Molecular – direct	25 mL
Leight et al. [88]	2018	USA	North America	High	38	Seawater	Molecular – direct	100 mL
Merga et al. [89]	2014	UK	Europe	High	9	Untreated influent of wastewater treatment facilities	-	-
Lee et al. [90]	2012	USA	North America	High	129	Freshwater from public beaches	-	200 mL
Giacometti et al. [91]	2015	Italy	Europe	High	30	-	Culture with enrichment	25 mL
Newton et al. [92]	2013	USA	North America	High	40	Influent from WWTP	Molecular – direct	200 mL
Acharya et al. [93]	2020	UK and Ethiopia	Europe/Africa	High/Low	9	River water, effluent from WWTP	Molecular – direct	100 mL/250 mL/ 500 mL/ 1 L
Niedermeyer et al. [94]	2020	Ethiopia	Africa	Low	96	Flood water	Culture with enrichment	50 mL filtered or 1.3 mL enrichment
Kutilova et al. [95]	2021	USA	North America	High	6	River water, inflow to hospital WWTP, raw hospital sewage, inflow to municipal WWTP	Molecular – direct	-
Godoy et al. [96]	2020	Czech Republic	Europe	High	12	River water	Molecular – direct	-
Zhang et al. [97]	2020	Brazil	South America	Upper middle	8	Effluent from WWTP	Molecular – direct	-
Miltenburg et al. [98]	2020	China	Asia	Upper middle	588	River water	Molecular - direct	-
Khan et al. [99]	2020	Canada	North America	High	173	Surface water (agricultural)	Molecular – direct	1 L
Zhang et al. [100]	2020	China	Asia	High	20	Coastal water, raw sewage	Molecular – direct	-

Table S2. Assessment of publication bias for the prevalence of *Arcobacteraceae* in aquatic environments using Egger's regression test.

**Outcome**	**Egger's Regression Test**			
	**95% CI**	***t***	**df**	***p*-value**
Prevalence of *Arcobacter* spp.	(0.817; 2.915)	3.547	70	<0.001

CI, confidence interval; df, degrees of freedom.
